# RNF2 Modulates Lipid Metabolism and Inflammation in Alcohol-associated Liver Disease by Interacting with USP7

**DOI:** 10.7150/ijbs.111290

**Published:** 2025-04-28

**Authors:** Qi Yan, Qi Fang, Zhiang Chen, Lijian Chen, Jian Du

**Affiliations:** 1 Department of Clinical Laboratory, the Second Affiliated Hospital of Anhui Medical University, China.; 2 Department of Biochemistry and Molecular Biology, Research Center for Infectious Diseases, School of Basic Medical Sciences, Anhui Medical University, Hefei 230032, China.; 3 Department of Anesthesiology, The First Affiliated Hospital of Anhui Medical University, Hefei 230032, China.; 4 Provincial Key Laboratory of Zoonoses of High Institutions in Anhui, Anhui Medical University, Hefei 230032, China.

**Keywords:** ALD, RNF2, lipid metabolism, PI3K/AKT

## Abstract

Alcohol-associated liver disease (ALD) is a widely prevalent chronic liver disease caused by alcohol overconsumption. However, the pathogenesis of ALD is complex and has not been fully elucidated. Ring finger protein 2 (RNF2) is associated with the occurrence and development of hepatocellular carcinoma (HCC), but its function in ALD has not been explored. In this study, we investigated the role of RNF2 in ALD and its underlying mechanisms. In vivo, an ALD model was established and adeno-associated virus (AAV8-shRNA-RNF2) was used to knock down RNF2. Liver injury, hepatic steatosis, and inflammation were assessed and functional studies were conducted in AML-12 cells and macrophages. The study found that hepatic-specific RNF2 knockdown attenuated EtOH-induced liver steatosis and inflammation. Furthermore, RNF2 knockdown significantly alleviated EtOH-mediated lipid accumulation and inflammation. Additionally, RNF2 interacted with ubiquitin-specific peptidase 7 (USP7) and regulated the phosphatidylinositol 3 kinase/protein kinase B (PI3K/AKT) signaling pathway. Importantly, inhibition of USP7 or PI3K/AKT signaling pathway suppressed lipid accumulation and inflammation in the ALD model. Our research demonstrated that RNF2 had a novel function of regulating lipid metabolism and inflammation in ALD through its interaction with USP7 and modulation of the PI3K/AKT signaling pathway.

## Introduction

Alcoholic beverages are common consumer goods in people's daily life. Nevertheless, long-term excessive drinking can lead to liver damage, commonly known as alcohol-associated liver disease (ALD). ALD often manifests as alcohol-related fatty liver in the early phase and may progress to alcoholic hepatitis, liver fibrosis, cirrhosis, or even hepatocellular carcinoma (HCC) 1. It is estimated that approximately 30% of HCC cases worldwide are caused by alcohol abuse 2. Compared to HCC from other etiologies, alcohol-associated HCC (A-HCC) is increasing in incidence and correlated with a worse prognosis 3. Several pharmacological or biological methods have been applied to treat ALD. However, new ideas and directions for treating ALD could be identified if new and more effective targets are discovered.

Hepatic steatosis is the earliest form of ALD characterized by excessive accumulation of lipids in the liver 4. Lipogenesis in the initial stage is considered an important risk factor for ALD progression. There is mounting evidence that EtOH upregulates the expression of sterol regulatory element binding protein 1c (SREBP-1c) and inhibits peroxisome proliferator-activated receptor α (PPAR-α) thus suppressing fatty acid oxidation and lipolysis and leading to further lipid accumulation 5, 6. In addition to steatosis, inflammation also plays a crucial role in the progression of ALD. The direct toxicity of EtOH and its metabolites disrupts the gut barrier, leading to intestinal dysregulation, which induces the expression of proinflammatory cytokines in the liver through the gut-liver axis 7, 8. In addition, lipid products that accumulate in hepatocytes lead to aggravated liver inflammation and injury 9. In brief, dysregulated lipid metabolism and inflammation contribute to subsequent liver damage and dysfunction in ALD. Therefore, effective treatments and new intervention targets through the regulation of lipid metabolism and inflammation are urgently needed to prevent ALD.

RNF2, also known as RING1b, is a member of the polycomb group (PcG) family and acts as the E3 ligase responsible for the mono-ubiquitination of histone H2A 10. Previous studies have reported that abnormal expression of RNF2 is involved in the occurrence and development of various types of cancers 11. Our group previously demonstrated that RNF2 promoted the progression of liver fibrosis via increasing inflammatory cytokines secretion, such as TNF-α, IL-1β and IL-6 12, In addition, inflammatory cytokines have been verified to play a crucial role in multiple liver diseases, including ALD and cirrhosis 13, 14. These suggested that RNF2 may modulate inflammation in alcohol-induced liver injury, as inflammation drives ALD progression 15. Moreover, the participation of RNF2 in liver diseases, including viral hepatitis and HCC, has also been reported in recent years 16, 17. Notably, RNF2 was highly expressed in HCC and was associated with malignant characteristics and poor prognosis of HCC 18. Coincidentally, patients with A-HCC tend to have a higher degree of malignancy and a poorer prognosis than those with HCC of other etiologies compared with patients with other etiologies of HCC 19, 20. Based on that, we hypothesized that RNF2 might play a critical role in the progression of ALD to HCC. Both liver fibrosis and HCC are further progressive forms of ALD. Given this consideration, RNF2 may participate in the development of ALD. However, studies on the relationship between RNF2 and ALD have not yet been reported.

This study aimed to explore the specific role of RNF2 in ALD and to elucidate the mechanism of RNF2 in lipid metabolism and the inflammatory response, identifying new potential ideas and targets for treating ALD. Furthermore, delivering interventions in the early stage of ALD is highly important for preventing further progression and reducing medical burden.

## Materials and methods

### Human liver tissues

Human liver samples were obtained from the First Affiliated Hospital of Anhui Medical University. The protocol was approved by the Ethics Committee of the First Affiliated Hospital of Anhui Medical University (20190214). ALD liver samples were collected from surgical resections of non-cancerous liver from ALD patients. Control liver samples were obtained from healthy areas of patients with hepatic hemangioma who underwent hepatectomy without fatty liver or hepatitis. Informed consent was obtained from each patient. The details about human liver samples are in Table 1.

### Reagents and materials

LY294002 (a PI3K inhibitor, HY-10108) was obtained from Med Chem Express (Shanghai, China). EtOH and controlled liquid diets were manufactured by Trophic Animal Feed High-Tech Co. Ltd (Nantong, China). Alanine aminotransferase (ALT) and aspartate aminotransferase (AST) detection kits were obtained from the Jiancheng Bioengineering Institute (Nanjing, China). RNF2 (16031-1-AP), TNF-α(17590-1-AP), IL-1β (66737-1-Ig) and USP7(66514-1-Ig) antibodies were purchased from Proteintech (Wuhan, China). IL-6 (WL02841) and PPAR-α (WL00978) were obtained from Wanleibio (Shenyang, China). ACOX-1(DF12046) antibody was purchased from Affinity Biosciences (USA). AKT (YT0185), p-AKT(YP0006) and SREBP-1c(YT6055), PI3K (YT6156), and p-PI3K (YP0224) polyclonal antibodies were purchased from Immunoway Biotechnology Company (USA). β-actin monoclonal antibody and HRP-conjugated secondary antibodies were obtained from Proteintech (Wuhan, China). Trizol and Lipofectamine^TM^3000 were purchased from Invitrogen (USA). The primers of RNF2, IL-6, IL-1β, TNF-α, PPAR-α, ACOX-1, SREBP-1c and β-actin were purchased from Tsingke Biotechnology Co., Ltd. (Beijing, China). Opti-MEM and fetal bovine serum (FBS) were obtained from Gibco (USA), and DMEM/F-12 medium was obtained from Cytiva (USA). RIPA lysis buffer and PMSF were purchased from Beyotime (Shanghai, China). An Oil Red O staining kit (G1262) was obtained from Solarbio Life Sciences (Beijing, China). The pEGFP-C2-RNF2 and pEGFP-C2 plasmids were preserved in our laboratory.

### Animal experiment

6-8-week-old male C57BL/6J mice were purchased from GemPharmatech Co., Ltd (Jiangsu, China). Mice were maintained in an environment with 20-25 °C, 50±5% relative humidity and a light /dark cycle for 12/12 h, and were fed a standard diet for 7 days before the experiments. According to the ALD model 21, the process of model establishment included a liquid diet adaptation period (5 d), a building period (10 d), and EtOH gavage for a single occurrence, which lasted for 16 days. The adeno-associated virus AAV8-shRNA-NC/RNF2 (pAAV-U6-shRNA(NC/RNF2)-CBh-EGFP-WPRE) was purchased from OBiO Technology Co., Ltd. (Shanghai, China). Mice were randomly divided into 4 groups as described below (n = 6). (1) Control group: the mice were fed a control liquid diet plus a single gavage of normal saline (5 g/kg) on the last day. (2) EtOH group: the mice were fed a 5% EtOH liquid diet during the building period plus a single gavage of EtOH (5 g/kg). (3) shNC EtOH group: the AAV8-shRNA-NC virus was injected into the tail vein to establish an RNF2 knockdown (KD) mouse control model, and after 1 month, the same modeling procedure was maintained as the EtOH group. (4) shRNF2 EtOH group: the AAV8-shRNA-RNF2 virus was injected into the tail vein to construct an RNF2 KD mouse model, and after 1 month, the same modeling procedure was maintained as the EtOH group. The liver tissues and serum were harvested for subsequent experiments after 9 h of the last EtOH gavage. All animal experiments were approved by the Health Medical Research Ethics Committee of Anhui Medical University (No. LLSC20190022).

### Serum aminotransferase activity

The blood samples were centrifuged at 4500 rpm for 15 min, and the serum was collected to measure the activity of ALT and AST.

### Hepatic triglyceride analysis

Liver tissues (100 mg) were mechanically homogenized in 0.9 mL of homogenate medium under the condition of an ice-cold water bath. The samples were centrifuged at 2500 rpm for 10 min. Supernatants were collected and liver triglycerides were analyzed using a triglyceride assay kit according to the manufacturer's protocols (Nanjing jiancheng Bioengineering Institute, China).

### Cell culture

The AML-12 cells were preserved in our laboratory. AML-12 cells were cultured in DMEM/F12 supplemented with 1% penicillin and streptomycin and 10% FBS in an incubator (37°C and 5% CO_2_). In addition, AML-12 cells were treated with distinct concentrations of EtOH (0, 25, 50, 100, 150, 200) mM. Finally, cells were stimulated for 24 h with 100 mM EtOH to establish the cell model of ALD.

### Plasmid transfection and RNA interference

AML-12 cells were uniformly inoculated in six-well plates. Then, the pEGFP-C2 or pEGFP-C2-RNF2 plasmid was transfected into AML-12 cells to overexpress RNF2 *via* Lipofectamine^TM^3000. In addition, the expression level of RNF2 was decreased by infecting with RNF2 knockdown lentivirus (RNF2-shRNA), and NC-shRNA was used as a negative control. Subsequently, AML-12 cells were co-cultured with or without EtOH for 24 h and were collected for later experiments.

### RNA extraction and quantitative reverse transcription-polymerase chain reaction (RT-qPCR)

Total RNA was extracted from AML-12 cells and liver tissues with TRIzol reagent (Invitrogen). Then, RNA was reverse-transcribed into cDNA. RT-qPCR was performed with a SYBR Green Premix Kit, after which relative mRNA expression was measured. The mRNA expression levels of target genes were normalized to that of β-actin, which was used as an internal control. The PCR primers used are displayed in Table 2.

### Western blotting

The protein samples were obtained from AML-12 cells and liver tissues using RIPA lysis buffer. Equivalent amounts of protein were separated *via* SDS-PAGE and subsequently transferred to PVDF membranes. After being blocked in 5% skim milk, the membranes were washed with TBST buffer 3 times and incubated with the corresponding primary antibodies at 4°C overnight. The dilution ratios used were as follows: RNF2 (1:1000), SREBP-1c (1:1000), TNF-α (1:1000), ACOX-1 (1:1000), IL-6 (1:1000), PPAR-α (1:1000), IL-1β (1:1000), USP7(1:1000), p-AKT (1:1000), AKT (1:1000), p-PI3K (1:1000), PI3K (1:1000) and β-actin (1:3000). Afterwards, the membranes were incubated with HRP-conjugated secondary antibodies for 1.5 h (at 1:8000 dilution). The protein bands were detected by an enhanced chemiluminescence (ECL) Kit after the membranes were washed.

### Hematoxylin-eosin (H&E) staining

To assess pathological changes in liver tissues, liver tissues were harvested, fixed in 4% paraformaldehyde, embedded in paraffin, and cut into sections (5 μm thick). The sections were stained with hematoxylin and eosin.

### Immunofluorescence

The expression and location of RNF2 and USP7 were detected in AML-12 cells. After fixation, the cells were blocked with 5% BSA before they were incubated with primary antibodies. Then, the cells were incubated with diluted RNF2 (1:200) and USP7 (1:200) antibodies at 4°C overnight. The appropriate fluorescent secondary antibodies were incubated for 1 h, after which DAPI was used to label the nuclei.

### Oil red O staining

The frozen liver tissues were sliced into 10 μm-thick sections and then stained with Oil Red O working solution at RT. Subsequently, the sections were washed 3 times with PBS and counterstained with hematoxylin. In addition, AML-12 cells were divided into a control group and an EtOH group. After the corresponding treatment, Oil Red O staining was performed according to the manufacturer's instructions.

### Co-IP

A co-immunoprecipitation assay was used to verify the interaction between RNF2 and USP7. Briefly, RNF2 or USP7 antibodies were conjugated to the protein A/G agarose beads. Then, the AML-12 cells were lysed with pre-cooled IP buffer, and the supernatants of cell lysates were incubated with beads conjugated with the corresponding antibodies for 4 h at 4°C. Finally, Western blotting analysis was used to test the interactions.

### RNA-seq

AML-12 cells were transfected with NC-shRNA or RNF2-shRNA and then cultured in medium containing 100 mM EtOH for 24 h. TRIzol reagent was used to lyse the AML-12 cells, after which the lysates were harvested for further RNA-seq analysis (Oebiotech, Shanghai, China). The crossover differentially expressed genes (DEGs, P < 0.05 and |log2FC| > 2.0) were subjected to Kyoto Encyclopedia of Genes and Genomes (KEGG) analysis.

### Statistical analysis

The cellular experiments were repeated in triplicate. At least 6 separate samples were subjected to biochemical analysis of the animals in each group. All the data are expressed as mean ± SD. The statistical significance of differences between two groups was assessed by unpaired *t*-test, whereas comparisons between multiple groups were performed by one-way ANOVA. A value of *P < 0.05, ** P < 0.01, *** P < 0.001 was considered to indicate statistical significance.

## Results

### RNF2 expression was increased in ALD

**Figure 1:**
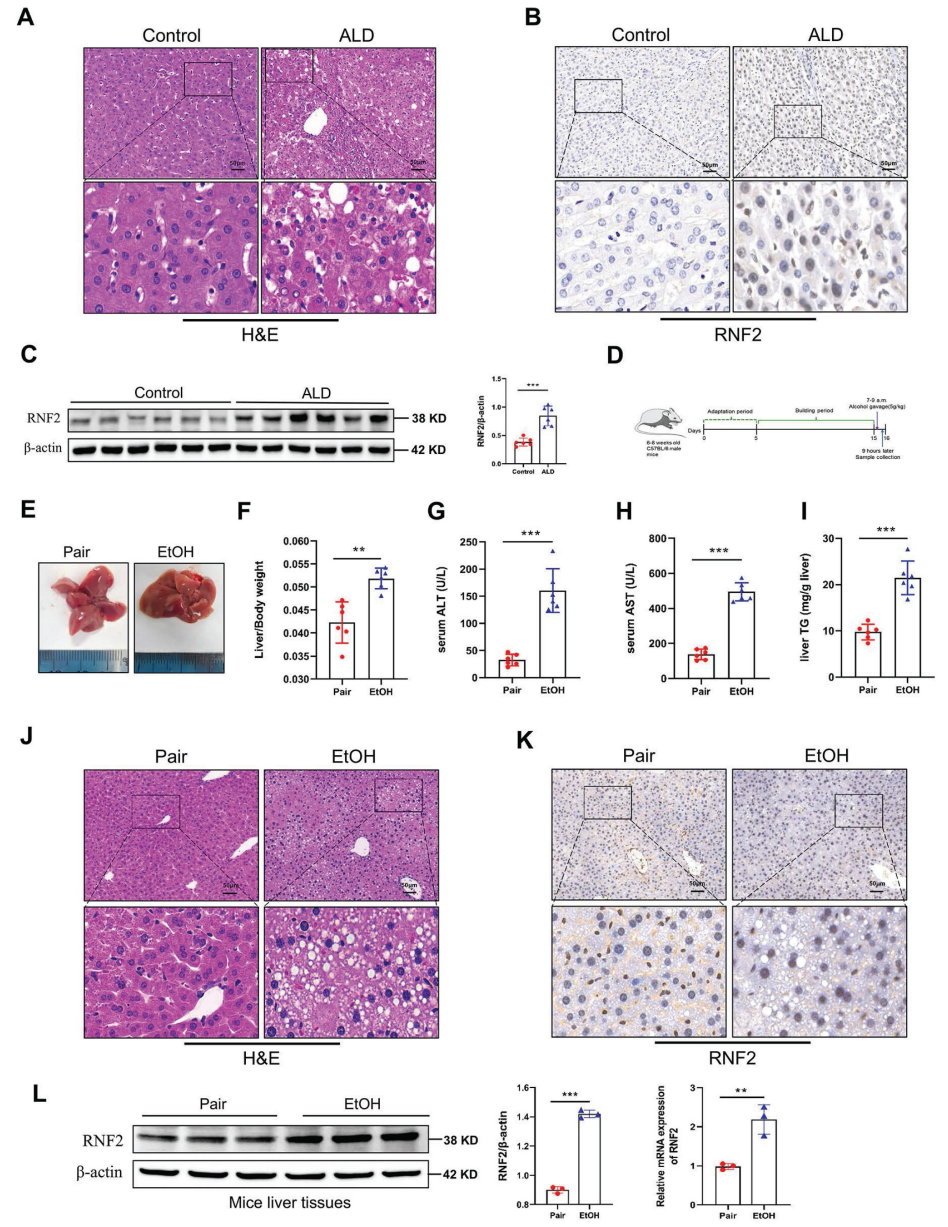
** RNF2 expression was increased in ALD.** A. H&E staining of ALD patients and healthy control donors.B. Immunohistochemistry (IHC) staining of RNF2 in the livers of ALD patients and healthy control donors. C. Western blotting and quantitative analysis of RNF2 expression in the livers of ALD patients and healthy control donors. D. Schematic diagram establishing the ALD model. E. Appearance of mouse livers in pair group and EtOH group. F. Liver-to-body weight ratio of mice in pair group and EtOH group (n=6/group). G, H, I. Determination of serum ALT, AST and hepatic TG levels in the pair group and EtOH group. J. H&E staining of liver tissue in the two groups. Scale bar:50 μm. K. IHC staining of RNF2 in EtOH-induced mouse livers. Scale bar:50 μm. L. RNF2 expression levels in the livers of mice in pair group and EtOH group.

We first collected liver tissues from ALD patients who were diagnosed with alcohol-associated hepatitis/cirrhosis. The pathological histology analysis showed that liver tissues in patients with ALD are characterized by fat vacuoles, disorganization of hepatocytes, and expansion of the cell space (Fig. 1A). Immunohistochemical staining and Western blotting results revealed that RNF2 expression was increased in ALD patients compared to healthy donors, and RNF2 was located in the nucleus (Fig. 1B, C). To investigate whether RNF2 is involved in ALD progression and its underlying mechanisms, ALD model mice were established according to the diagram (Fig. 1D). It was intuitively seen that the livers of ALD model mice were more yellowish than those of the pair group (Fig. 1E). Additionally, the liver-to-body weight ratio was greater in EtOH group than in the pair group (Fig. 1F), as well as the serum ALT, AST and hepatic triglyceride (TG) levels (Fig. 1G-I), suggesting that the liver was damaged. The extent of liver injury was evaluated by histopathological analysis. H&E staining revealed that a normal liver had little steatosis, and liver lobules were arranged in an orderly manner around the central vein. In contrast, mice in the EtOH group exhibited fat vacuoles, intercellular space expansion and disordered arrangement of hepatocytes (Fig. 1J). The immunohistochemistry results suggested that RNF2 expression was upregulated in ALD mice (Fig. 1K). Furthermore, RT-qPCR and Western blotting revealed that the RNF2 expression level was elevated in the EtOH group compared to the pair group (Fig. 1L). These data showed that RNF2 expression was increased in ALD mice and that abnormal RNF2 expression might be related to the progression of ALD.

### EtOH induced lipid accumulation and inflammation response in the livers of ALD mice

Fat vacuoles were clearly observed in the liver tissues by H&E staining. To further detect lipid accumulation, Oil Red O staining was performed. The results exhibited significant lipid accumulation and hepatic steatosis in ALD model mice compared with pair-fed mice, as indicated by the presence of red lipid droplets (Fig. S1A). In addition, transmission electron microscopy (TEM) was used to observe the ultrastructure of the hepatocytes. There were many lipid droplets in the hepatocytes, and the mitochondria appeared to be damaged, with a loss of mitochondrial cristae and altered mitochondrial morphology (Fig. S1C). The expression levels of lipid metabolism-related genes (PPAR-α, ACOX-1 and SREBP-1c) in the liver were also examined, and the results showed that SREBP-1c expression was increased, while the expression of PPAR-α and ACOX-1 was decreased in ALD mice compared to those in the pair group (Fig. S1B). It is well known that EtOH may induce inflammation during liver injury. The expression levels of inflammatory cytokines, including IL-1β, TNF-α, and IL-6, in the serum were detected. Consistent with the findings of previous studies, the serum IL-1β, TNF-α, and IL-6 levels were increased in ALD mice (Fig. S1D-F). In addition, the immunohistochemical staining results suggested that the number of infiltrating macrophages and neutrophils was increased in the liver tissues of ALD mice (Fig. S1G).

Similarly, the protein expression levels of TNF-α, IL-6, and IL-1β were also increased in the liver tissues of the ALD group (Fig. S1H). In brief, EtOH feeding not only caused lipid accumulation but also induced inflammation in ALD model.

### RNF2 was upregulated in EtOH-induced AML-12 cells

**Figure 2:**
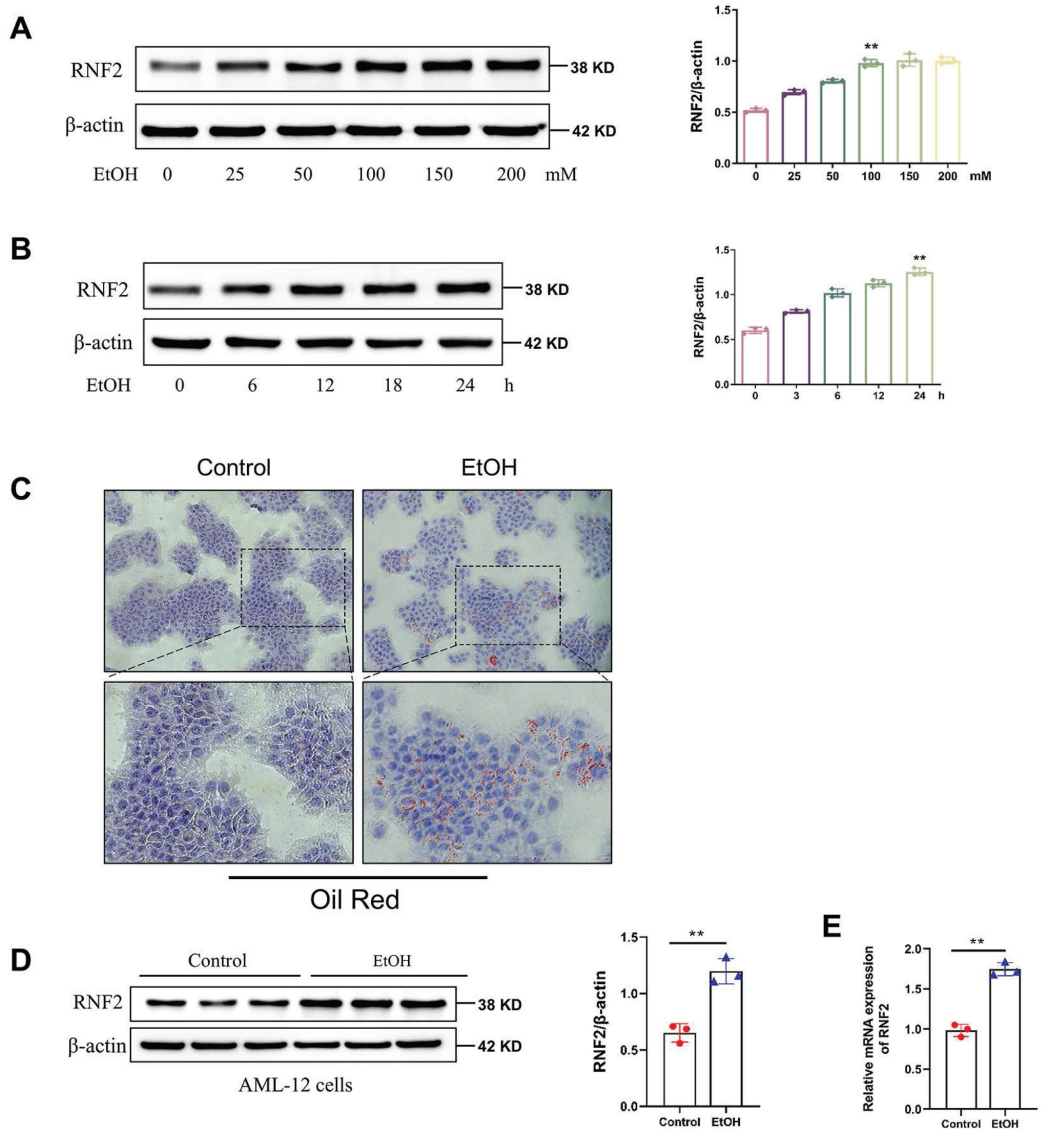
** RNF2 was upregulated in EtOH-induced AML-12 cells.** A. AML-12 cells were pretreated with different concentrations of EtOH (0, 25, 50, 100, 150, or 200 mM), after which RNF2 protein expression was measured. B. RNF2 protein expression peaked after stimulation for 24 h with 100 mM EtOH. C. Oil Red O staining of EtOH-induced AML-12 cells. The cells were treated with EtOH (100 mM) for 24 h. D, E. RNF2 mRNA and protein expression levels. All the results were obtained from three independent experiments.

To investigate the alteration in the expression of RNF2, we detected the expression of RNF2 in EtOH-induced AML-12 cells at different times and concentrations. First, AML-12 cells were stimulated with 0, 25, 50, 100, 150, or 200 mM of EtOH, and Western blotting was used to measure RNF2 expression. The results suggested that RNF2 expression increased in a dose-dependent manner and peaked at 100 mM (Fig. 2A). Next, after different treatment durations, RNF2 expression peaked after continuous stimulation for 24 h with 100 mM EtOH (Fig. 2B). Oil Red O staining was performed after stimulation with 100 mM EtOH for 24 h, and the results suggested that EtOH induced the production of many red lipid droplets (Fig. 2C). These findings indicated that the* in vitro* ALD model was successfully constructed in EtOH-treated AML-12 cells. The results of RT-qPCR and Western blotting verified that the RNF2 expression level was significantly increased after EtOH treatment (Fig. 2D-E). Moreover, we established the RNF2 overexpression or knockdown model to examine the effect of RNF2 alteration. The pEGFP-C2-RNF2, pEGFP-C2, RNF2-shRNA and NC-shRNA were transfected into AML-12 cells, respectively. Notably, RNF2 expression was increased after EtOH treatment, while pEGFP-C2-RNF2 further upregulated RNF2 expression (Fig. S2A). In contrast, infection with RNF2-shRNA caused a significant decrease in RNF2 mRNA and protein levels, despite stimulation with EtOH (Fig. S2B). These data suggested that RNF2 was upregulated in EtOH-induced AML-12 cells.

### RNF2 silencing reduced lipid accumulation in EtOH-induced AML-12 cells

**Figure 3:**
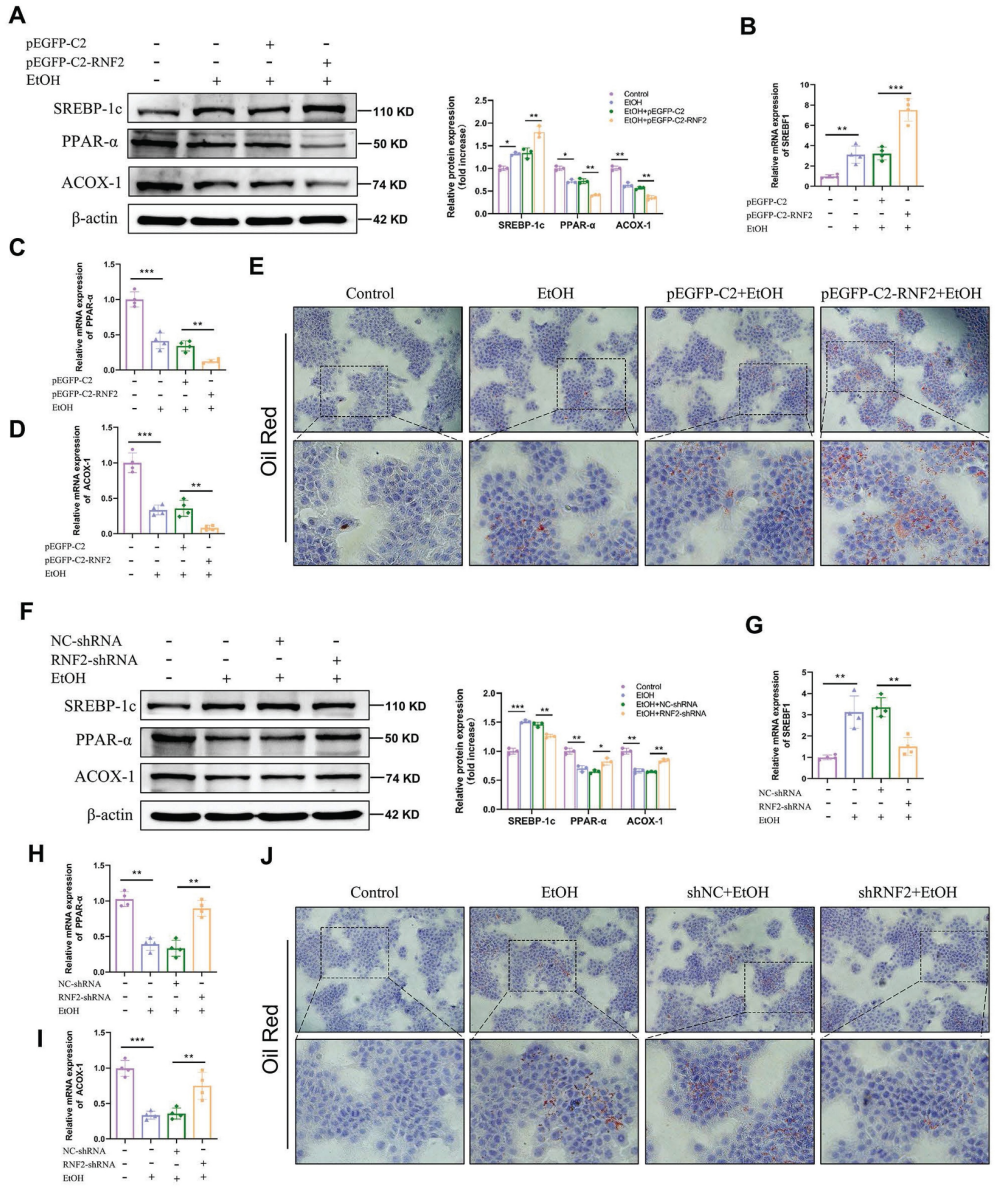
** RNF2 silencing reduced lipid accumulation in EtOH-induced AML-12 cells.** A-D. The mRNA and protein expression levels of PPAR-α, ACOX-1 and SREBP-1c in pEGFP-C2- or pEGFP-C2-RNF2-transfected and EtOH-induced AML-12 cells. E. Oil Red O staining of EtOH-induced AML-12 cells with RNF2 overexpression. F-I. The protein and mRNA expression levels of PPAR-α, ACOX-1 and SREBP-1c were measured in NC-shRNA- or RNF2-shRNA-infected and EtOH-induced AML-12 cells. J. Oil Red O staining of EtOH-induced AML-12 cells with RNF2 knockdown.

To determine the influence of RNF2 on lipid accumulation, the expression levels of lipid metabolism-related genes (SREBP-1c, PPAR-α and ACOX-1) were measured in RNF2-overexpressing or RNF2-knockdown AML-12 cells. RT-qPCR and Western blotting indicated that the expression levels of PPAR-α and ACOX-1 were decreased in the EtOH group compared with the control group. Nonetheless, the expression of SREBP-1c, one of the transcriptional regulators of lipid synthesis genes, was markedly increased. Besides, overexpression of RNF2 further exacerbated this phenomenon (Fig. 3A-D). The Oil Red O staining was used to detect lipid accumulation. Results suggested that EtOH treatment increased lipid accumulation, and RNF2 overexpression further aggravated it (Fig. 3E). Notably, this phenomenon was reversed when RNF2 was silenced. Compared with those in the NC-shRNA group, the expression of SREBP-1c was reduced, and PPAR-α and ACOX-1 were upregulated (Fig. 3F-I). And the number of lipid droplets decreased significantly in the RNF2 knockdown AML-12 cells (Fig. 3J). In brief, the above results suggested that RNF2 knockdown reduced lipid accumulation in EtOH-induced AML-12 cells.

### RNF2 promoted the inflammatory response in EtOH-induced macrophages

**Figure 4:**
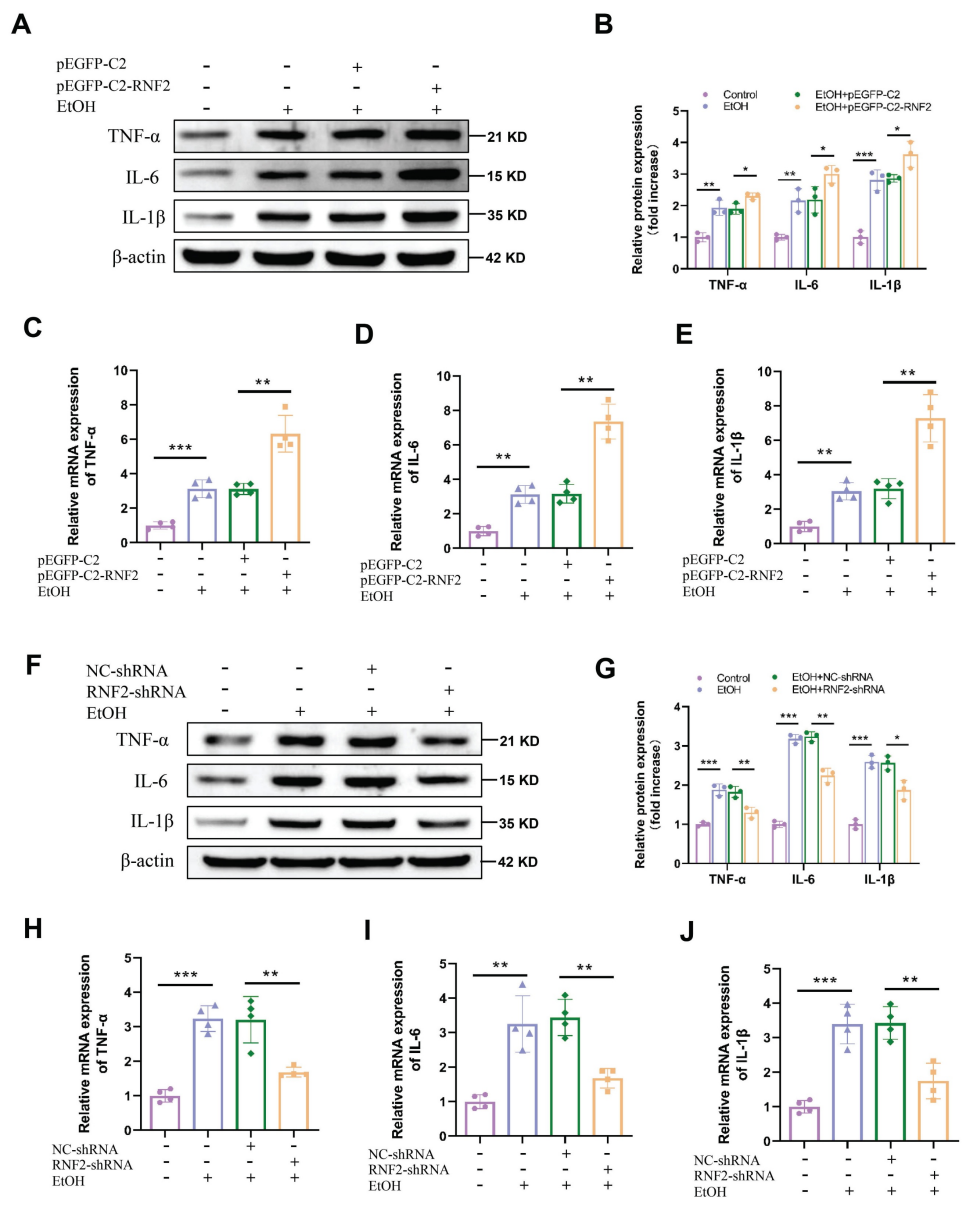
** RNF2 promoted inflammatory response in EtOH-induced RAW264.7 cells.** A-B. Western blotting and quantitative analysis of TNF-α, IL-6 and IL-1β expression in pEGFP-C2- or pEGFP-C2-RNF2-transfected and EtOH-induced RAW264.7 cells. C-E. The mRNA expression levels of TNF-α, IL-6 and IL-1β. F-G. Western blotting and quantitative analysis of TNF-α, IL-6 and IL-1β expression in NC-shRNA- or RNF2-shRNA-infected and EtOH-induced RAW264.7 cells. H-J. The mRNA and expression levels of TNF-α, IL-6 and IL-1β.

Next, we aimed to determine whether RNF2 affects the EtOH-induced inflammatory response in macrophages. As mentioned earlier, RNF2 was overexpressed or knocked down by transfecting pEGFP-C2-RNF2 or RNF2-shRNA, respectively. Subsequently, cells were stimulated with EtOH for 24 h, and the expression of inflammation-related genes was tested. Inflammation response was significantly increased after EtOH stimulation, manifested by upregulation of the mRNA and protein expression levels of TNF-α, IL-6, and IL-1β. Meanwhile, overexpression of RNF2 further enhanced inflammation induced by EtOH in macrophages, and increased expression of TNF-α, IL-6, and IL-1β (Fig. 4A-E). Conversely, knockdown of RNF2 decreased the expression of TNF-α, IL-6, and IL-1β at both the mRNA and protein levels (Fig. 4F-J). In short, EtOH stimulation induced an inflammatory response in macrophages, whereas RNF2 silencing inhibited the expression of inflammation-related genes.

### RNF2 regulated lipid accumulation and the inflammatory response via the PI3K/AKT signaling pathway

**Figure 5:**
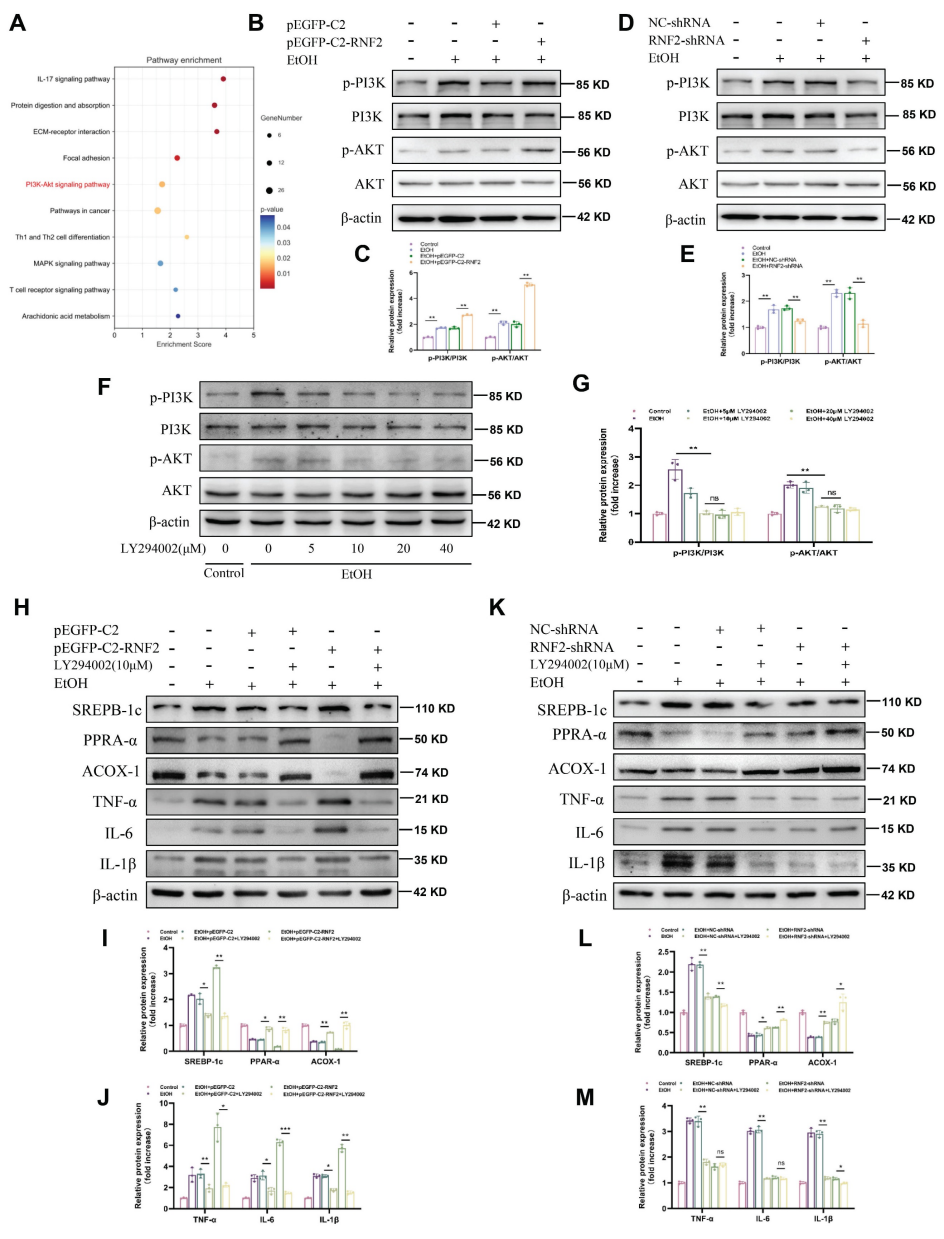
** RNF2 regulated lipid accumulation and the inflammatory response via the PI3K/AKT signaling pathway.** A. KEGG enrichment analysis of DEGs in NC-shRNA- or RNF2-shRNA-infected AML-12 cells treated with EtOH. B-C. The protein expression of p-PI3K and p-AKT in pEGFP-C2- or pEGFP-C2-RNF2-transfected and EtOH-induced AML-12 cells. D-E. The protein expression of p-PI3K and p-AKT in NC-shRNA or RNF2-shRNA infected and EtOH-induced AML-12 cells. F-G. AML-12 cells were incubated with LY294002 (0, 5, 10, 20, 40) μM for 24 h, and protein levels of p-PI3K and p-AKT were detected to determine the appropriate concentration. H-J. The relative protein expression levels of PPAR-α, ACOX-1, SREBP-1c, IL-1β, TNF-α, and IL-6 were detected by Western blotting in pEGFP-C2- or pEGFP-C2-RNF2-transfected AML-12 cells, with or without LY294002 treatment. K-M.The relative protein expression of PPAR-α, ACOX-1, SREBP-1c, IL-1β, TNF-α, and IL-6 were detected by Western blotting in NC-shRNA- or RNF2-shRNA-infected AML-12 cells, with or without LY294002 treatment.

To elucidate the mechanism through which RNF2 affected ALD, we performed an RNA sequencing (RNA-seq) analysis of hepatocytes treated with EtOH and infected with RNF2-shRNA or NC-shRNA. KEGG pathway analysis of the DEGs revealed that the PI3K/AKT signaling pathway was likely involved in the regulation of EtOH-induced AML-12 cells (Fig. 5A). We detected the levels of p-PI3K and p-AKT to determine the role of RNF2 in the PI3K/AKT signaling pathway. The results suggested that EtOH treatment increased p-PI3K and p-AKT expression in AML-12 cells, and RNF2 overexpression further activated the PI3K/AKT signaling pathway (Fig. 5B-C). In contrast, RNF2 knockdown significantly inhibited the increase in p-PI3K and p-AKT in EtOH-induced AML-12 cells (Fig. 5D-E). Then, LY294002 (the PI3K inhibitor) was subsequently used to examine the effect of the PI3K/AKT signaling pathway. Cells were pretreated with different concentrations of LY294002, and the result suggested that 10 μM is an appropriate concentration to inhibit the PI3K/AKT signaling pathway (Fig. 5F-G). We next investigated whether silencing of RNF2 alleviated the inflammatory response and lipid accumulation in EtOH-induced AML-12 cells by inhibiting the PI3K/AKT signaling pathway. First, AML-12 cells were generated by overexpressing or knocking down RNF2 *via* the corresponding plasmids, after which the cells were cultured with LY294002 for 24 h. After treatment with EtOH, the expression levels of lipid metabolism-related and inflammation-related proteins were detected. The results showed that, compared with those in the pEGFP-C2 group, PPAR-α and ACOX-1 expression in the pEGFP-C2+LY294002 group was increased, whereas the expression of SREBP-1c was inhibited. The expression of inflammation-related genes, including TNF-α, IL-6, and IL-1β, were also suppressed by LY294002. Although the overexpression of RNF2 aggravated lipid accumulation and inflammation, LY294002 still reversed the effect induced by RNF2 overexpression in EtOH-induced AML-12 cells (Fig. 5H-J). In contrast, compared with those in the RNF2-shRNA group, the expression levels of SREBP-1c, PPAR-α, ACOX-1, IL-1β, TNF-α, and IL-6 in the RNF2-shRNA+ LY294002 group did not significantly change (Fig. 5K-M). Taken together, these findings suggested that RNF2 modulated lipid accumulation and inflammatory response through the PI3K/AKT signaling pathway.

### RNF2 interacted with ubiquitin-specific peptidase 7 (USP7) and activated the PI3K/AKT signaling pathway

**Figure 6:**
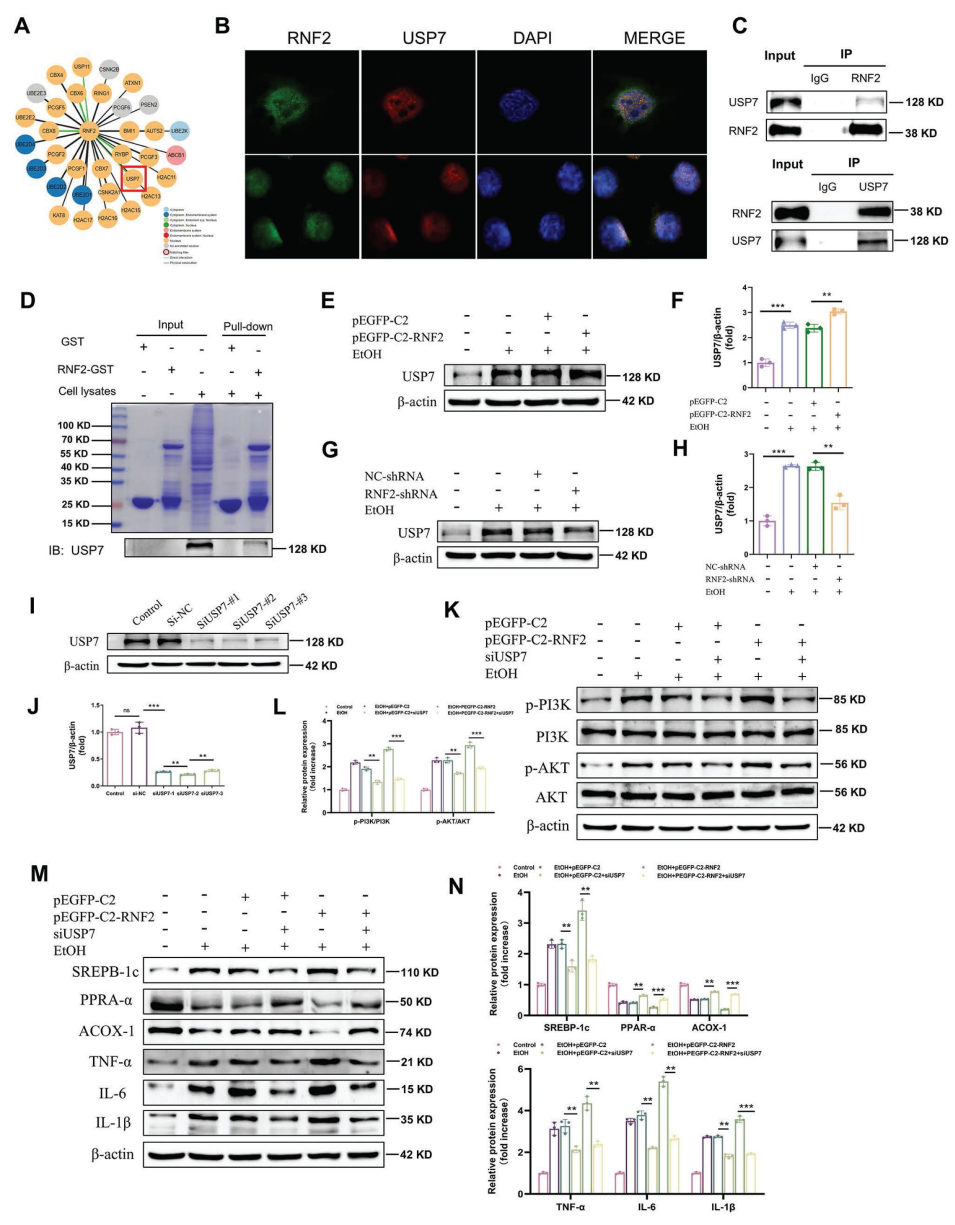
** RNF2 interacted with USP7 and activated the PI3K/AKT signaling pathway.** A. The prediction of RNF2-binding proteins in the STING database. B. Double-labeling immunofluorescence revealed a nuclear co-localization of RNF2 and USP7 proteins in AML-12 cells. C. The interaction between RNF2 and USP7 was tested by co-IP. D. The interaction between RNF2 and USP7 was tested by GST pull-down, and the purified GST protein was used as the control. E-H. Effect of RNF2 overexpression or knockdown on USP7 expression. I-J. Verification of the utility of USP7-siRNA. K-L. Effect of USP7-siRNA on the PI3K/AKT signaling pathway. M-N. Effect of USP7-siRNA on the expression of lipid metabolism-related and inflammation-related genes.

**Figure 7:**
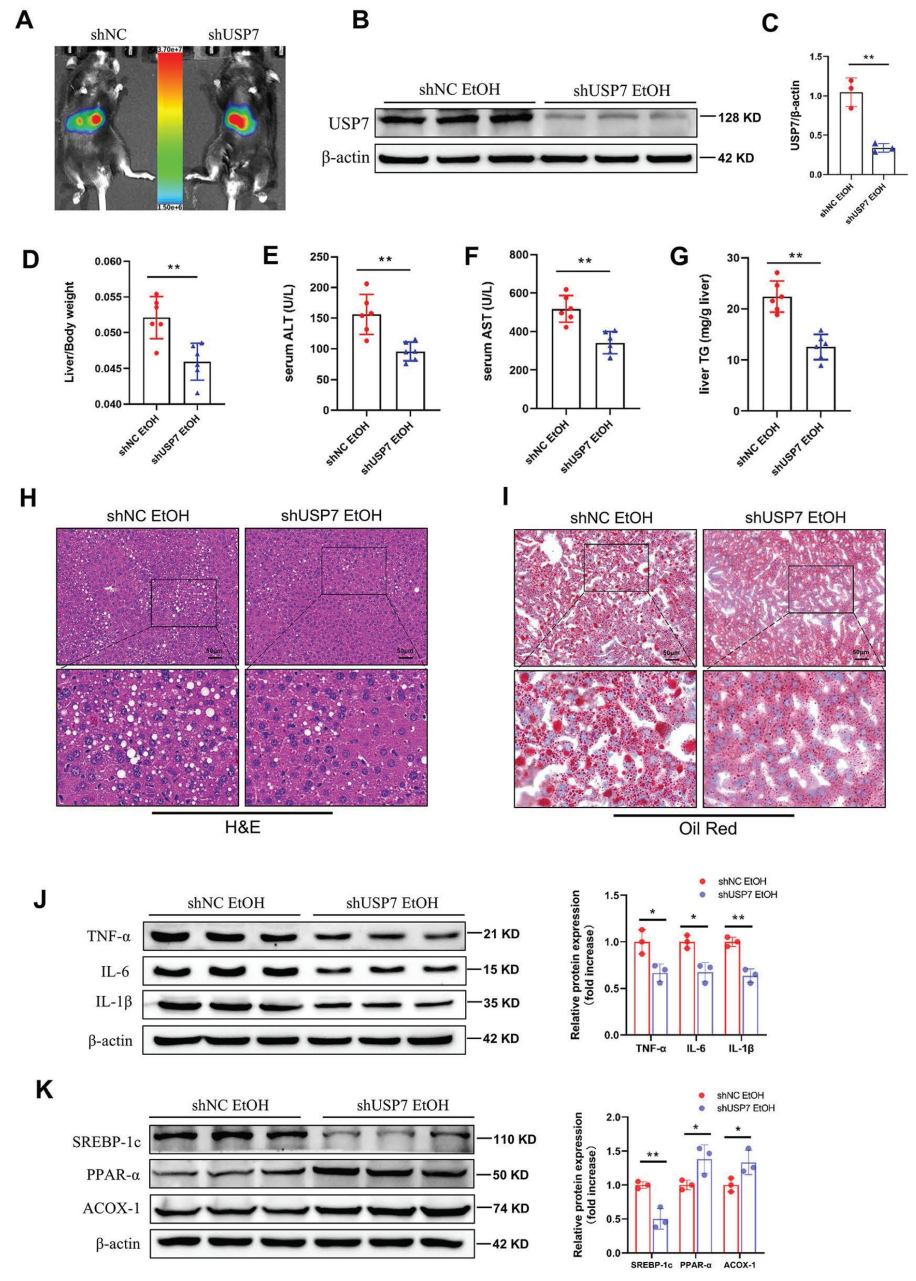
**Function of USP7 in ALD model mice.** A. Analysis of small animal imaging. B-C. Western blotting and quantitative analysis of USP7. D. Liver-to-body weight ratios of mice in the shNC EtOH group and shUSP7 EtOH group (n=6/group). E-G. Determination of the serum ALT, AST and hepatic TG levels in the shNC EtOH group and shUSP7 EtOH group. H. H&E staining of liver tissues from the two groups. Scale bar:50 μm. I. Oil Red O staining. J. The relative protein expression levels of TNF-α, IL-6 and IL-1β were detected by Western blotting in liver tissues of shNC EtOH group and shUSP7 EtOH group. K. The relative protein expression levels of PPAR-α, ACOX-1 and SREBP-1c in liver tissues from the shNC EtOH group and shUSP7 EtOH group.

Mechanistically, RNF2 was predicted to interact with USP7 according to the STING database (Fig.6A). Immunofluorescence colocalization revealed that RNF2 and USP7 colocalized in the nucleus (Fig. 6B). In addition, co-IP studies revealed an interaction between endogenous RNF2 and USP7 (Fig.6C). Moreover, endogenous USP7 was efficiently pulled down by the GST-tagged RNF2 protein (Fig. 6D). Interestingly, RNF2 overexpression increased the expression of USP7, while RNF2 silencing suppressed USP7 expression (Fig. 6E-H). Therefore, USP7-siRNA was used to interfere with the expression of USP7 for the following experiments (Fig. 6I-J). As shown in Fig. 6K-L, p-PI3K and p-AKT expression was increased after EtOH treatment, but USP7-siRNA suppressed p-PI3K and p-AKT despite the overexpression of RNF2. Consistently, USP7-siRNA suppressed the expression of SREBP-1c and inflammation-related genes (TNF-α, IL-6 and IL-1β) while increasing the expression of PPAR-α and ACOX-1 (Fig. 6M-N). To further explore the function of USP7 in ALD, USP7 was silenced by injecting AAV8-shRNA-USP7 into the tail vein of mice (Fig. 7A-C). The mice in the shUSP7 group exhibited lower liver-to-body weight ratio, serum ALT and AST, and hepatic TG levels compared to the shNC group (Fig. 7D-G). Meanwhile, the liver injury and steatosis of mice were alleviated in the shUSP7 group (Fig. 7H-I). And the protein expression levels of TNF-α, IL-6, IL-1β, and SREBP-1c were decreased while ACOX-1 and PPAR-α were increased in the shUSP7 group (Fig. 7J-K). In summary, these data demonstrated that RNF2 interacted with USP7 and modulated the PI3K/AKT signaling pathway in EtOH-induced AML-12 cells. Additionally, USP7 knockdown reduced hepatic steatosis and liver injury in ALD mice *in vivo*.

### RNF2 knockdown attenuated liver injury in ALD model mice

**Figure 8:**
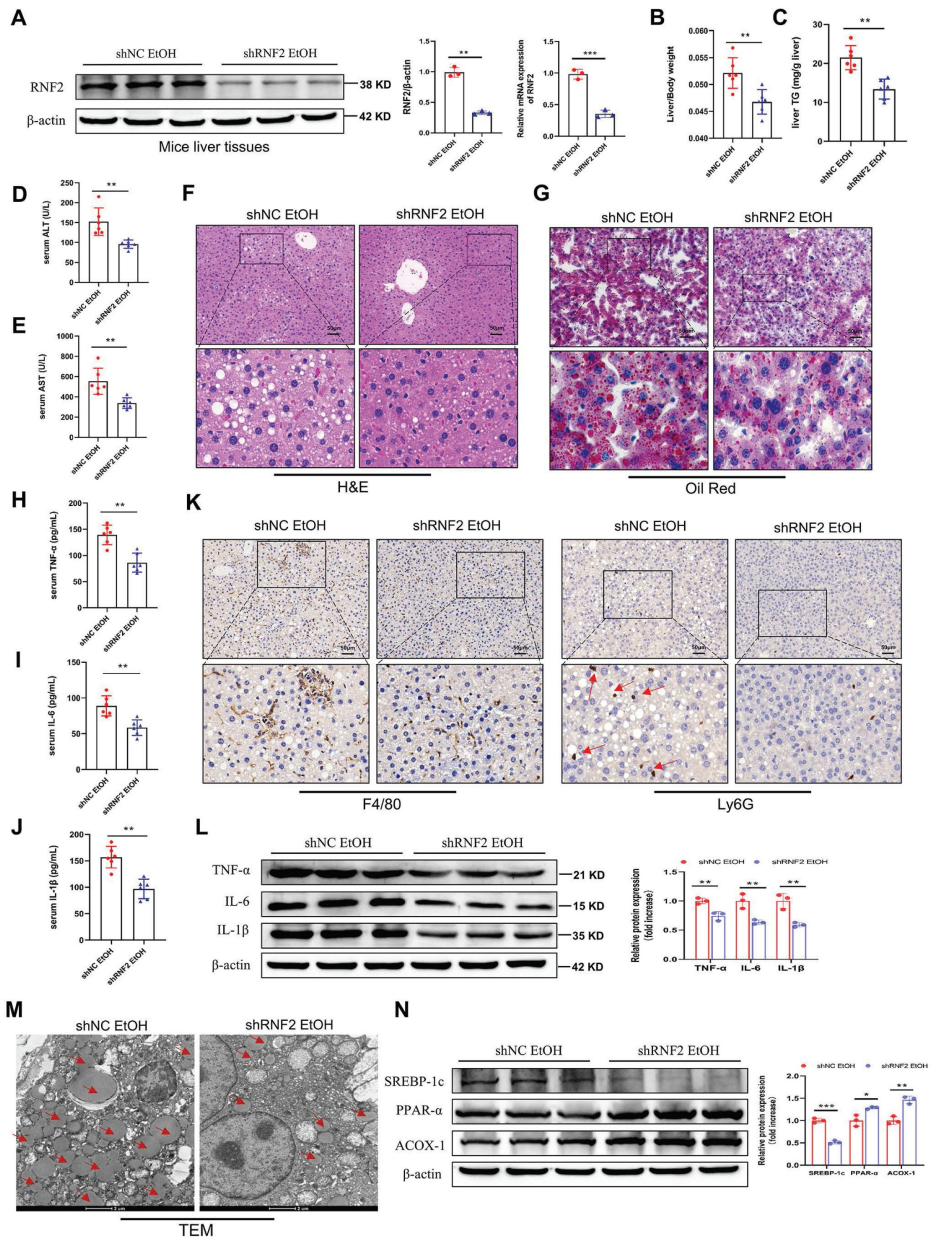
**RNF2 knockdown attenuated liver injury in ALD model mice.** A.The mRNA and protein expression levels of RNF2. B. Liver-to-body weight ratios of mice in the shNC EtOH group and shRNF2 EtOH group (n=6/group). C-E. Determination of the serum ALT, AST and hepatic TG levels in the shNC EtOH group and shRNF2 EtOH group. F. H&E staining of liver tissues from the two groups. Scale bar:50 μm. G. Oil Red O staining. H-J. Serum levels of TNF-α, IL-6 and IL-1β in the shNC EtOH group and shRNF2 EtOH group. K. IHC staining of F4/80 and Ly6G in the livers of mice in shNC EtOH group and shRNF2 EtOH group. L. The relative protein expression levels of TNF-α, IL-6 and IL-1β were detected by Western blotting in liver tissues of shNC EtOH group and shRNF2 EtOH group. M. The changes in intracellular structure were observed via TEM. The arrow points to lipid droplets. N. The relative protein expression levels of PPAR-α, ACOX-1 and SREBP-1c in liver tissues from the shNC EtOH group and shRNF2 EtOH group.

To determine the function of RNF2 *in vivo*, the adeno-associated virus AAV8-shRNA-RNF2 and its control vector were injected into mice to silence RNF2 (Figure S3A). The fluorescence image confirmed that AAV8-shRNA-RNF2 was successfully located in the liver and was expressed (Figure S3B). Simultaneously, RNF2 was downregulated at both the mRNA and protein levels in the shRNF2-treated group (Figure 8A). Additionally, H&E staining manifested that the degree of liver injury and steatosis were attenuated in the shRNF2 group (Figure 8F). The serum ALT, AST and hepatic TG levels were also lower in the shRNF2 group than in the shNC group (Figure 8C-E), as well as the liver-to-body weight ratio (Figure 8B). The levels of proinflammatory cytokines in the serum were also examined. Serum levels of TNF-α, IL-6, and IL-1β were significantly decreased after RNF2 knockdown (Figure 8H-J), consistent with the trend of protein expression levels in the mouse liver (Figure 8L). Meanwhile, the immunohistochemical staining results also suggested that the number of infiltrating macrophages and neutrophils decreased significantly after RNF2 knockdown (Figure 8K). The results of Oil Red O staining indicated less hepatic steatosis in the shRNF2 group than in the shNC group (Figure 8G). Moreover, fewer lipid droplets and less severely damaged mitochondria were observed in the hepatocytes by TEM (Figure 8M). Similarly, Western blotting revealed that the expression of ACOX-1 and PPAR-α was upregulated, while the expression of SREBP-1c was decreased in the shRNF2 group (Figure 8N). In summary, RNF2 knockdown reduced hepatic steatosis and inflammation, and attenuated liver injury in ALD mice.

## Discussion

ALD is a widely prevalent liver disease resulting from chronic or binge consumption of alcohol. Liver is the major organ involved in alcohol metabolism and is therefore susceptible to alcohol intake. Notably, epidemiological trends predicted an increased burden of ALD, and ALD was a main cause of liver-related morbidity and mortality 22. Hence, it is pivotal to discover novel therapeutic strategies for treating ALD. In the ALD model, EtOH induced the release of free fatty acids (FFAs) and increased TG levels in the circulatory system. Then excess FFAs were transported to the liver, leading to hepatic lipid accumulation 23. In addition, the liver can secrete various inflammatory cytokines including IL-1β, IL-6 and TNF-α because it contains multiple immune cells, such as NK cells, T cells, neutrophils, and macrophages, which promoted the progression of ALD 24. Indeed, the complex pathogenesis of ALD was associated with the toxic effects of EtOH and its metabolites, the inflammatory response and so on. Moreover, lipid metabolism disorders may occur when pro-inflammatory cytokines are increased 25. Thus, targeting inflammatory-lipid metabolism to alleviate EtOH-induced liver injury is a potential strategy for treating ALD. The present study showed that EtOH feeding obviously induced liver injury, inflammation, and steatosis. Notably, RNF2 knockdown ameliorated liver pathology progression in ALD model mice. Previous studies and our group have reported that RNF2 is involved in the occurrence and development of multiple liver diseases, including liver fibrosis, viral hepatitis, and HCC 12, 16, 17. However, there have not been any studies between RNF2 and ALD, especially concerning its specific regulatory mechanism.

In the present study, RNF2 expression was upregulated in ALD patients. Similarly, RNF2 was also increased in the livers of ALD model mice, and this change was accompanied by liver steatosis and injury. On this basis, we further explored whether RNF2 affected lipid metabolism. Lipid accumulation was one of the early features of ALD and controlling the development of steatosis may effectively alleviate disease progression 4. Hence, genes involved in regulating lipid metabolism, such as PPAR-α, ACOX-1, and SREBP-1c, were also closely related to ALD. SREBP-1c was a crucial transcriptional regulator of lipid biosynthesis, and its overexpression decreased lipid oxidation and increased lipid synthesis, thereby causing lipid accumulation in hepatocytes 26. PPAR-α was widely expressed in the liver and maintained liver and whole-body fatty acid homeostasis. Deletion of PPAR-α impaired fatty acid catabolism, leading to the worsening of hepatic steatosis 27. In addition, PPAR-α regulated the expression of ACOX-1, which was involved in fatty acid β-oxidation as a key rate-limiting enzyme 28. Our data indicated that RNF2 knockdown alleviated liver injury and hepatic steatosis in ALD model mice. Moreover, silencing of RNF2 reduced lipid accumulation by inhibiting SREBP-1c expression and increasing PPAR-α and ACOX-1 expression in EtOH-induced AML-12 cells. Additionally, we further explored the effect of RNF2 on the inflammatory response. These results suggested that EtOH induced hepatic inflammation and increased the release of pro-inflammatory cytokines* in vivo*, while RNF2 knockdown inhibited these responses. RT-qPCR and Western blotting indicated that inhibition of RNF2 decreased the expression of inflammatory cytokines in EtOH-treated AML-12 cells. According to the above results, RNF2 was involved in regulating ALD progression through at least two aspects—inflammation and lipid metabolism.

Mechanistically, Kyoto Encyclopedia of Genes and Genomes (KEGG) pathway analysis suggested that RNF2 was closely related to the PI3K/AKT signaling pathway, which was reported to correlate with hepatic lipid metabolism homeostasis 29. Moreover, PI3K/AKT signaling pathway inhibitors regulated lipid metabolism by rescuing EtOH-mediated increases in lipogenic genes (SREBP-1c and FASN) and reducing fatty acid oxidation genes (PPAR-α and ACOX-1) in EtOH-treated primary mouse hepatocytes 30. Moreover, activation of the PI3K/AKT signaling pathway induced by hepatic phosphoenolpyruvate carboxykinase 1 (PCK1) deficiency stimulates lipid synthesis. Silencing of AKT1 or treatment with AKT inhibitors alleviated metabolic-associated fatty liver disease progression *in vivo* 31. However, no reports on the relationship between RNF2 and the PI3K/AKT signaling pathway have been published, and further investigations are needed. EtOH treatment activated the PI3K/AKT signaling pathway, and RNF2 overexpression in EtOH-treated AML-12 cells increased the phosphorylation of PI3K and AKT, whereas RNF2 knockdown dampened these events. Moreover, LY294002 (a PI3K inhibitor) alleviated lipid synthesis and the inflammatory response *via* inhibition of the PI3K/AKT signaling pathway. The effects of RNF2 on the PI3K/AKT signaling pathway were consistent with previous findings. Given that the PI3K/AKT signaling pathway was widely considered an important lipid metabolism mediator, the RNF2-PI3K/AKT signaling pathway may partly contribute to lipid accumulation and inflammation in ALD.

Additionally, among the most important findings of this study was the identification of USP7 as an interaction target of RNF2. USP7 is an essential deubiquitinating enzyme (DUB) in all eukaryotes and is involved in multiple biological processes, including viral infection and tumorigenesis 32, 33. Previous studies have reported that USP7 promoted the progression of hepatoblastoma, and melanoma through the PI3K/AKT signaling pathway 34. Similarly, we observed parallel increase in USP7 and RNF2 expression in EtOH-treated AML-12 cells, resulting in the activation of the PI3K/AKT signaling pathway. However, RNF2 knockdown was accompanied by decreased USP7 expression. This finding led us to hypothesize that USP7 might be involved in RNF2 function. Indeed, USP7 silencing inhibited the activation of the PI3K/AKT signaling pathway and subsequent lipid accumulation and inflammation. In addition, the detailed mechanisms of RNF2 and USP7 were still unclear. A possible explanation was that RNF2 may regulate USP7 expression as a transcriptional regulatory factor 35. The E3 ligase RNF220 was reported to stabilize β-catenin instead of promoting its ubiquitination and proteasomal degradation to promote the Wnt signaling pathway 36. Therefore, RNF2, a member of the RING finger protein (RNF) family, may also play a role by stabilizing USP7 to regulate the PI3K/AKT signaling pathway. In ALD, our data showed that USP7 knockdown ameliorated liver injury and metabolic dysfunction, suggesting that targeting USP7 could potentially provide a more precise treatment for ALD. It had been reported that USP7 inhibitors (GNE-6776, P5091, etc.) were widely used in various diseases. GNE-6776 was explored for the treatment of breast cancer 37, non-small cell lung cancer 38, and Epstein-Barr virus infection 39. P5091 was also used for multiple diseases such as melanoma 40 and inflammatory bowel disease 41. Our study complemented the existing research by addressing the role of USP7 in the field of ALD. The application of USP7 inhibitors for ALD therapy based on pre-existing safety data provides a clinical translation strategy to bridge preclinical validation and human trials. Collectively, the continuous exploration and discovery of therapeutic targets such as USP7, will expand the therapeutic options and possibilities for ALD treatment. It will provide novel perspectives and promising strategies for ALD treatment, as well as the target RNF2 explored in this study.

In this study, we proposed that RNF2 was involved in lipid accumulation and inflammation in ALD. Although our study focused on ALD, the role of RNF2 in lipid metabolism and inflammation suggests its potential relevance in Metabolic Dysfunction-Associated Steatotic Liver Disease (MASLD). MASLD affects approximately 30% of the global population, with its prevalence steadily increasing 42, which has a broader impact than ALD. MASLD and ALD share overlapping pathological, molecular mechanisms, and clinical features despite differing etiologies. Both MASLD and ALD are characterized by excessive lipid accumulation, chronic inflammation, and even progress to fibrosis and cirrhosis 43. Our data demonstrated that RNF2 modulated lipid metabolism and inflammation in ALD via PI3K/AKT signaling pathway. Considering that PI3K/AKT signaling pathway is also the core pathogenesis of MASLD 44, 45, RNF2 may similarly influence lipid homeostasis and inflammatory responses in MASLD. Future studies will focus on whether RNF2 is involved in MASLD and whether its expression change affects disease phenotypes. This cross-disease relevance highlights RNF2 as a potential therapeutic target for multiple forms of Steatotic Liver Disease.

## Conclusions

In brief, the data indicated the significant role of RNF2 in the progression of ALD. RNF2 expression was upregulated in ALD and inhibition of RNF2 alleviated lipid accumulation and the inflammatory response. Mechanistically, RNF2 interacted with USP7 and activated the PI3K/AKT signaling pathway to regulate ALD progression. These findings provided a novel perspective for specifically targeting RNF2 in the field of ALD therapy. In the future, more advanced methods will be used to investigate the underlying mechanisms of RNF2 in ALD, facilitating the development of promising therapies.

## Supplementary Material

Supplementary figures and tables.

## Figures and Tables

**Table 1:** Characteristics of Control and ALD patients

	Age (years)	Gender	Drinking number of years	Drinking frequency	Clinic Diagnosis
Control 1	28	Male	0	Occasional	None
Control 2	59	Female	0	N/A	None
Control 3	66	Female	0	N/A	None
Control 4	53	Male	0	Occasional	None
Control 5	35	Male	0	Occasional	None
Control 6	32	Female	0	N/A	None
ALD patient 1	48	Male	30	Heavy	Cirrhosis
ALD patient 2	80	Male	45	Heavy	Cirrhosis/HCC
ALD patient 3	65	Male	42	Heavy	Cirrhosis
ALD patient 4	60	Male	32	Heavy	Cirrhosis/steatohepatitis
ALD patient 5	65	Male	28	Heavy	Cirrhosis
ALD patient 6	48	Male	16	Heavy	Cirrhosis

**Table 2:** Primer sequences

Genes	Sequences	
SREBF1	F: GATGTGCGAACTGGACACAG	R: CATAGGGGGCGTCAAACAG
PPAR-α	F: AGAGCCCCATCTGTCCTCTC	R: ACTGGTAGTCTGCAAAACCAAA
ACOX-1	F: TCCAGACTTCCAACATGAGGA	R: CTGGGCGTAGGTGCCAATTA
TNF-α	F: CCTCTCTCTAATCAGCCCTCTG	R: GAGGACCTGGGAGTAGATGAG
IL-6	F: ACTCACCTCTTCAGAACGAATTG	R: CCATCTTTGGAAGGTTCAGGTTG
IL-1β	F: ATGATGGCTTATTACAGTGGCAA	R: GTCGGAGATTCGTAGCTGGA
RNF2	F: GAGTTACAACGAACACCTCAGG	R: CAATCCGCGCAAAACCGATG
β-actin	F: GGCTGTATTCCCCTCCATCG	R: CCAGTTGGTAACAATGCCATGT

## Abbreviations

ALD: alcohol-associated liver disease
HCC: hepatocellular carcinoma
RNF2: ring finger protein 2
USP7: ubiquitin-specific peptidase 7
PRC1: polycomb repressive complex 1
SREBP-1c: sterol regulatory element binding protein 1c
PPAR-α: peroxisome proliferator-activated receptor α
PcG: polycomb group
ALT: alanine aminotransferase
AST: aspartate aminotransferase
TNF-α: tumor necrosis factor-α
IL-6: interleukin-6
IL-1β: interleukin-1β
RT-qPCR: reverse transcription-quantitative polymerase chain reaction
TG: triglyceride
TEM: transmission electron microscope
FFA: free fatty acid
DUB: deubiquitinating enzyme
PI3K: phosphatidylinositol 3 kinase
AKT: protein kinase B
EtOH: ethanol
AAV: adeno-associated virus
H&E: hematoxylin and eosin
KEGG: kyoto Encyclopedia of Genes and Genomes
DEG: differentially expressed gene
