# Supplementary material for: RNF2 Modulates Lipid Metabolism and Inflammation in Alcohol-associated Liver Disease by Interacting with USP7
Source: Int J Biol Sci. 2025 Apr 28;21(7):3306–23. doi: 10.7150/ijbs.111290 (PMC12080400; doi:10.7150/ijbs.111290)
Supplement: Supplementary file 1 — Supplementary figures and tables. [file ijbsv21p3306s1.pdf]

# Fig. S1

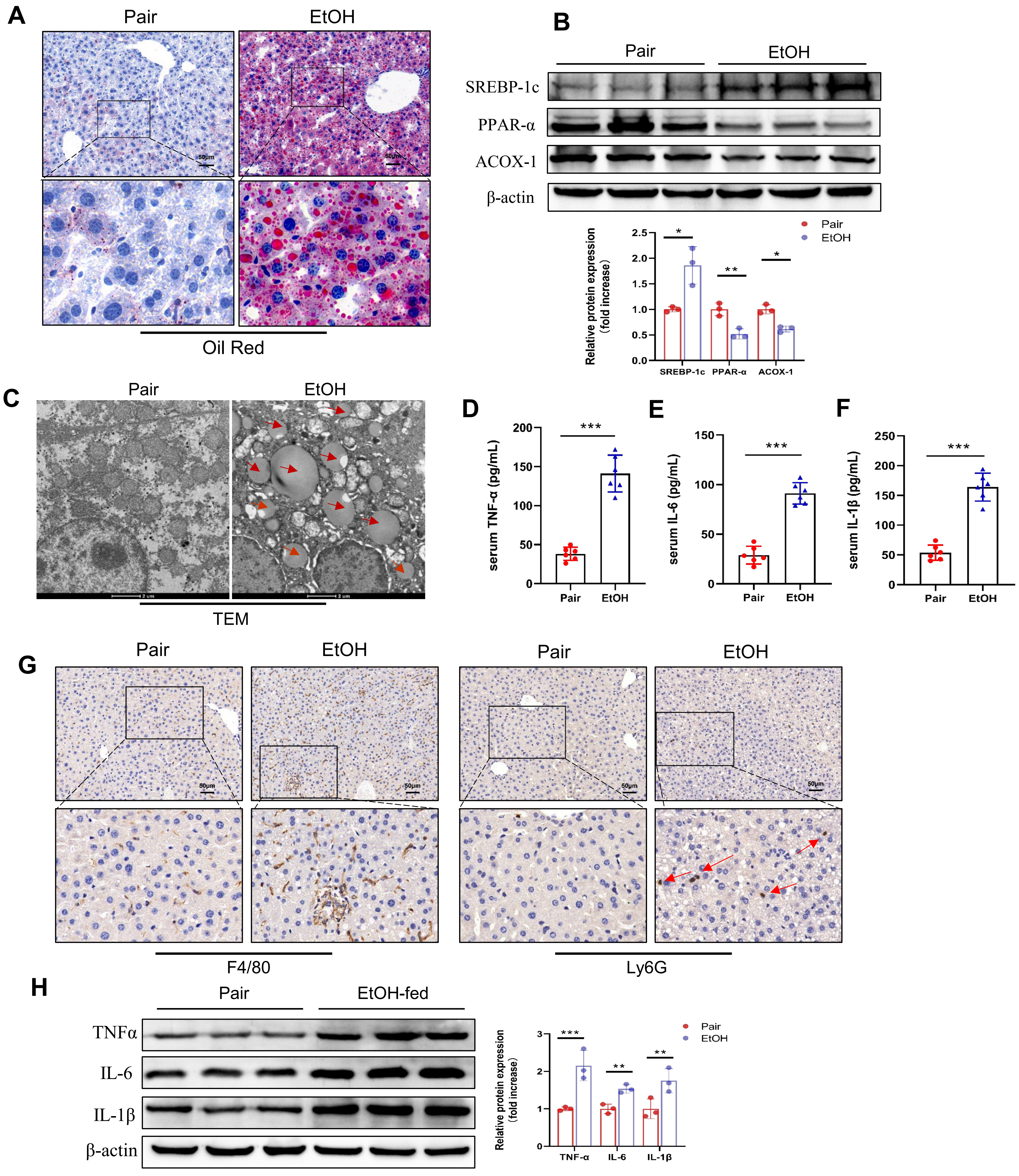


**Figure S1. EtOH induced lipid accumulation and an inflammatory response in the livers of ALD mice.** A. Frozen liver sections were stained with Oil Red O. B. Protein expression levels of PPAR-α, ACOX-1 and SREBP-1c in mouse liver tissues. C. Changes in the intracellular structure were observed via TEM. The arrow points to lipid droplets. D, E, F. Serum levels of IL-1β, TNF-α and IL-6 in pair group and EtOH group. G. IHC staining of F4/80 and Ly6G in the livers of mice in pair group and EtOH group. H. Protein expression levels of IL-1β, TNF-α and IL-6 in mouse liver tissues. The experiments were performed three times independently. **P* < 0.05, ** *P* < 0.01, *** *P* < 0.001 compared with the pair group..

# Fig. S2

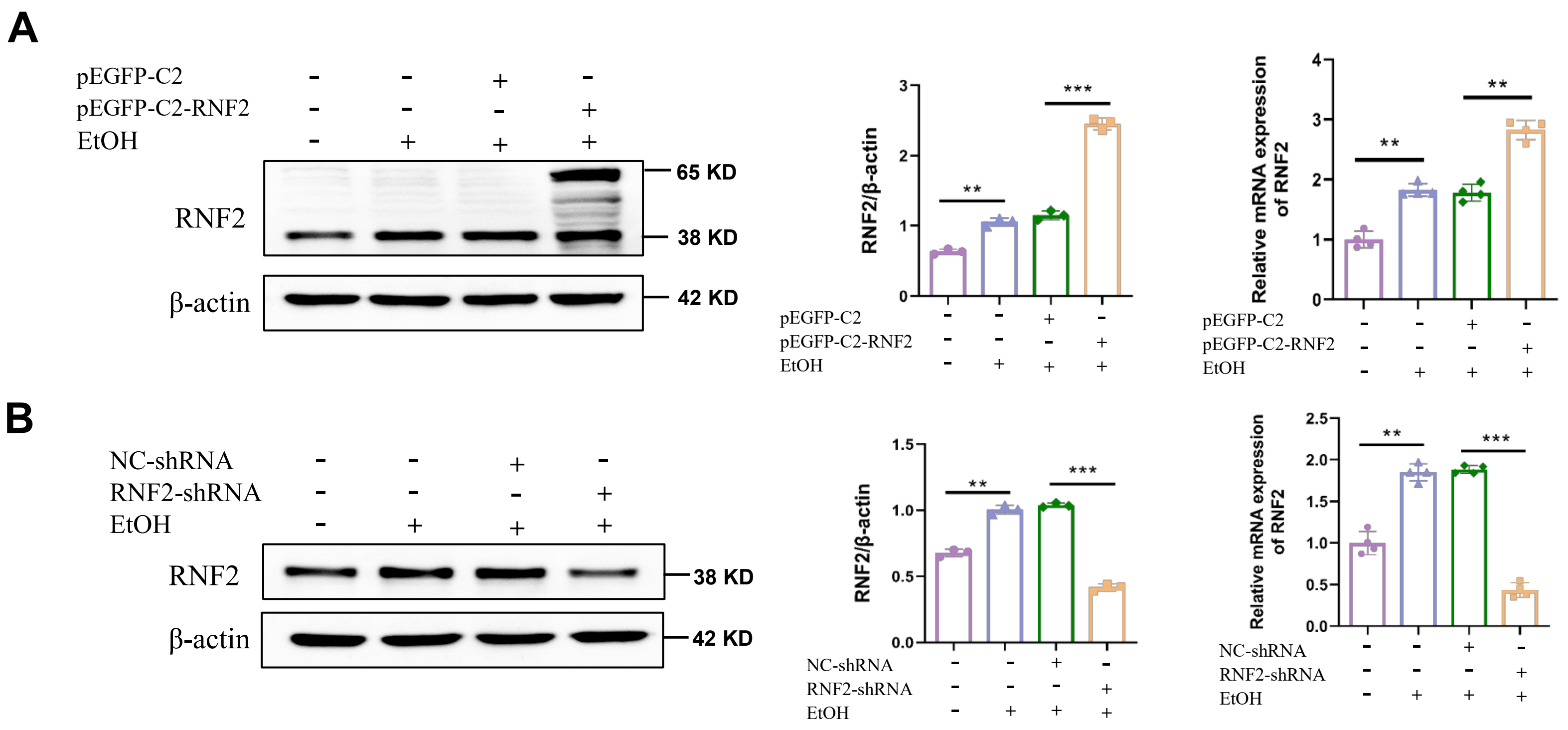


**Figure S2. The efficiency of RNF2 overexpression and knockdown.**

**A**. The mRNA and protein expression levels of RNF2 in pEGFP-C2- or pEGFP-C2-RNF2-transfected AML-12 cells treated with EtOH.

B. The protein and mRNA expression levels of RNF2 in NC-shRNA- or RNF2-shRNA-infected AML-12 cells treated with EtOH.

## Fig. S3

A

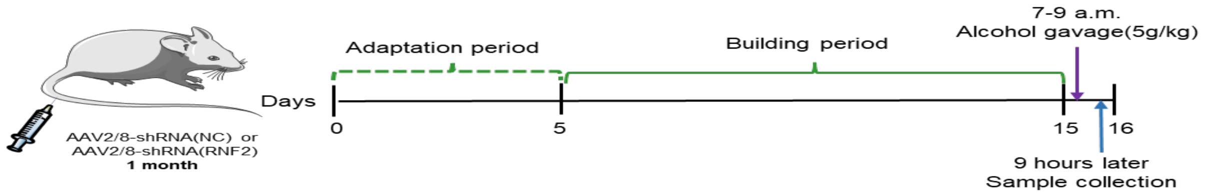


B

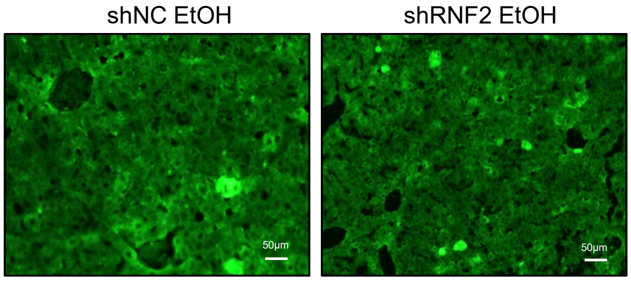


A. Schematic of the model construction process.
B. Fluorescence imaging confirmed that AAV8-shRNA (RNF2) was successfully localized to the liver and was successfully expressed.
